# *Catostylus tagi*: partial rDNA sequencing and characterisation of nematocyte structures using two improvements in jellyfish sample preparation

**DOI:** 10.1186/s40409-015-0037-4

**Published:** 2015-10-05

**Authors:** Tiago Parracho, Zilda Morais

**Affiliations:** Centro de Investigação Interdisciplinar Egas Moniz (CiiEM), Egas Moniz Cooperativa de Ensino Superior, Quinta da Granja, Monte de Caparica 2829-511 Caparica, Portugal

**Keywords:** Genomic DNA extraction, 18S 28S ITS1 ribosomal DNA, Structural macromolecules of nematocytes, *Catostylus tagi* jellyfish

## Abstract

**Background:**

More than 200 Scyphozoa species have been described, but few have been properly studied regarding their chemical and genetic characteristics. *Catostylus tagi*, an edible Scyphozoa and the sole European Catostylidae, occurs in summer at Tagus and Sado estuaries. Neither a systematic comparison between the two *Catostylus* communities nor a chemical approach on their nematocytes had been carried out yet.

**Methods:**

In order to achieve these purposes, optimisation of DNA extraction and of histochemical staining procedures were developed. *Catostylus* specimens from Tagus and Sado estuaries were compared by ribosomal 18S, 28S, and ITS1 partial sequencing. The morphochemistry of nematocytes was studied by optical and electronic microscopy.

**Results:**

Macroscopic and molecular results indicated that both communities belong to the same species, *C. tagi*. The hematoxylin and eosin staining allowed the visualisation of nematocyst genesis and indicated a basic character for the macromolecules on the shaft of euryteles and on the tubule of isorhizae and birhopaloids. By Masson’s trichrome procedure, the basic properties of the tubules were confirmed and a collagenous profile for the toxins was suggested. Results of the alcian blue staining showed that the outer membrane of nematocyte may consist of macromolecules with acidic polysaccharides, consistent with NOWA and nematogalectin glycoproteins detected in *Hydra*, but also with poly-gamma-glutamate complex, chitin-like polysaccharides and hyaluronic acids. Through the von Kossa assays, calcium was detected; its position suggested interactions with polysaccharides of the membrane, with proteins of the contractile system or with both.

**Conclusions:**

The optimisation of sample preparation for DNA extraction may facilitate further studies on little known jellyfish species. The improvement of the smear procedure simplified the use of stained reactions in zooplankton. Moreover, it was shown that good slide images might be acquired manually. The development of specific reactions, with traditional dyes and others, can give important contributions to clarify the chemical nature of the components of nematocytes. The characterisation of nematocyst toxins by staining tests is a goal to achieve.

## Background

Interest in marine zooplankton, mainly cnidarians, has increased as the current climate changes apparently support its expansion [1]. Cnidaria is an exclusively aquatic phylum, mostly marine, composed by six classes, of which the Anthozoa lives as sessile polyps while the other five classes (Hydrozoa, Scyphozoa, Staurozoa, Cubozoa and Polypodiozoa) usually have a life cycle with polyp and medusa stages, therefore forming the lineage of Medusozoa [2].

The organisms of a phylum often share phenotypic characters that are unique to the phylum. In the case of cnidarians, the primary commonality is the occurrence of cnidocyte (mostly known as nematocyte), a specialized cell which synthesizes an organelle named cnidocyst (usually referred as nematocyst). The nematocyst consists of a capsule with a coiled tubule inside containing toxins. Under stimulation, the capsule opens, through the operculum, and the tubule and toxins are rapidly ejected to outside, in attack or defense actions. Presently about 30 types of nematocysts are recognized, mainly based on Weill’s morphological classification of the tubule and the capsule [3].

In despite of the progress, data about cnidarians at a molecular level are still surprisingly scarce. Considering only scyphozoans, known as true jellyfish, more than 200 species have been described; however, only a few have been adequately studied in relation to the genetic characteristics and chemical composition of nematocytes [4, 5].

Regarding genetics, Bayha et al. [6] have proposed the analysis of jellyfish based on 18S and 28S ribosomal DNA. The relationships among 48 species representing 19 scyphozoan families were estimated using maximum likelihood and Bayesian phylogenetic methods.

Regarding the chemical composition, nematocyte components, both structural and toxins, are generally referred to have a peptide-like profile. The recent works on *Cyanea capillata* [7] and *Stomolophus meleagris* [8] allowed a better understanding on the nature and action of scyphozoan toxins, although large chemical variations are known to exist according to the species and geographical occurrence. In relation to structural compounds of nematocytes, as far as we know, the systematic studies on the morphochemistry of these complex organelles have been based on *Hydra* [9–17], which are hydrozoans. Therefore, it is necessary to develop techniques that contribute to information about scyphozoans.

The present study proposes two optimisation procedures, one for genomic DNA extraction and another one for the histochemical staining of some structural compounds of nematocytes, which were applied to the scyphozoan *Catostylus tagi* (Haeckel, 1869), the sole European specie of Catostylidae family. Medusae of *Catostylus * genus occur in summer at Tagus and Sado estuaries, the adult specimens exhibit a bell diameter around 25 cm and an average weight of 2.5 kg [18]. Occasional blooms of this edible jellyfish affect fishermen and bathers, since the venom causes skin rash accompanied by light pain for about 40 min [19], which is similar to the effect of Mediterranean scyphozoan [20]. To our knowledge, neither a systematic comparison between the two *Catostylus* communities nor an approach on their nematocyte compounds had been performed yet.

## Methods

Reagents were at least of analytical grade. Water was ASTM type II (Elix 10, Millipore, USA), hereafter referred to as water.

### Collecting samples

Jellyfish samples were collected within 500 m of the coordinates 38°41’00.9“N, 9°13’17.4” W, for Tagus samples, and 38°28’30.1“N, 8°50’54.5”W, for Sado samples, in several occasions between 2010 and 2013, in the period from July to September. *Catostylus* samples were caught with a fishing landing net in a motor boat and stored in isothermal icebox for a maximum of 3 h. Only adult animals with umbrella diameters between 19 and 27 cm were collected. At the laboratory, each animal was divided in three parts, namely gonads, oral arms and umbrella. After washing with water, each part was treated separately: gonads for DNA studies, oral arms and bell margins for morphochemistry, and umbrella without margins for other studies, reported elsewhere.

### Genetics

#### Sample preparation and DNA extraction

Gonads, with an approximate weight of 300 g, were fractionated using a dialysis membrane with nominal MWCO 15000 (Cellu Sep H1® 1-1550-45, USA). The process was carried out on a 2 L column full of water at 4 °C, with magnetic agitation, for 5 days, the water was swapped three times. The fraction of higher molecular weight was lyophilized until dryness (Modulyod-230, Thermo Electron Corp., USA). The extraction of DNA followed E.Z.N.A.® Mollusc DNA kit procedure (Omega Bio-tek, USA).

#### Amplification and sequencing of rDNA fragments

The primers for ITS1 rDNA were based on Dawson [21] while 18S and 28S rDNA primers were based on Bayha et al. [6]. The PCR program cycler (MJ Mini, Bio-Rad, USA) was optimized to 1× at 95 °C for 15 min and 35 cycles at 94 °C for 30 seconds; 60 °C for 15 s; 70 °C at 30 s; 72 °C for 6 min and ending at 4 °C. The amplified PCR fragments were purified on MicroSpin G-50 Sephadex™ (GE Healthcare, UK). The sequences of bases were obtained in a 3730xl DNA Analyzer (Applied Biosystems, USA) equipment, using BigDye® Terminator v3.1 Cycle Sequencing kit, with quality pattern Phred = Q > 20, in the STAB VIDA laboratory [22].

#### Comparison sequence

**Fig. 1:**
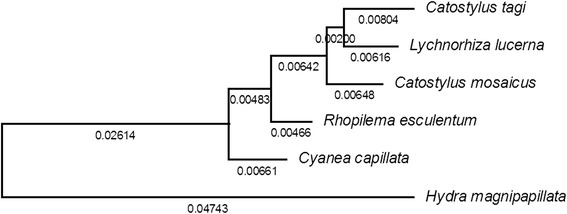
Cladogram based on 18S rDNA of *C. tagi, L. lucerna, C. mosaicus, R. esculentum, C. capillata* and *H. magnipapillata*

The homology was tested by Basic Local Alignment Search Tool, BLASTn option [23]; the sequences were introduced in FASTA format; the alignment of C. tagi sequences from Tagus and Sado was done through Megablast option, then obtaining the maximum percentage of identity. The consensus sequence of *C. tagi* from Tagus and Sado was acquired by Codoncode Aligner version 4.2.3 software. The 18S rDNA phylogenetic tree of was made with Molecular Evolutionary Genetics Analysis, Mega 5.1 beta three version [24]. Briefly, the sequences of other cnidarians, namely *C. mosaicus* [GenBank: HM194779.1], *L. lucerna* [GenBank: HM194807.1], *C. capillata* [GenBank: HM194820.1], *R. esculentum* [GenBank: HM194794.4] and *H. magnipapillata* [GenBank: EF059942.1], were obtained through the GenBank Nucleotide Database [25]. Together with *C. tagi* consensus sequence, the sequences were aligned by the Clustal method and the evolutionary history was inferred using the maximum composite likelihood (MCL) method, based on the Jukes-Cantor model. Cladograms were drawn automatically to scale, with branch length indicating the number of substitutions per site (Fig. 1).

### Morphology and morphochemistry of nematocytes

#### Scanning electron microscopy (SEM)

**Fig. 2:**
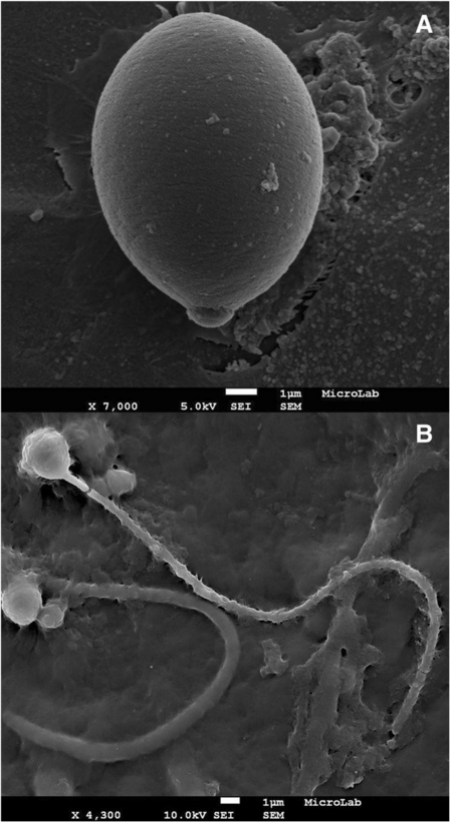
Nematocytes of *C. tagi* viewed through SEM. **a** Intact cell. **b** After nematocyst discharge; possibly an atrichous isorhiza (*left*) and a birhopaloid (*right*). Bars: 1 μm

Sample preparation of undischarged nematocysts (intact nematocytes): 1.0 g of tissue from the margins of oral arms of a jellyfish stored in formaldehyde 4 % were smashed in a porcelain mortar and left at ultrasound bath (Branson 2200, USA) in 30 mL ethanol 100 % for 15 min; then, 1 mL of the suspension was diluted in 20 mL ethanol 70 % and treated for SEM (Fig. 2a).

Sample preparation of discharged nematocysts (nematocytes with opened operculum): after smashing, sample was transferred to a 125 mL ultracentrifuge flask completed with ethanol 70 % and centrifuged (Beckman Coulter Optima LE-80 K, USA) at 20,000 g for 30 min at 4 °C; the resulting suspension was diluted as above (Fig. 2b).

Observations were performed with a HitachiS-2400 microscope (Japan), in MicroLab IST/UL [26], based on the procedure described by Peach and Pitt [27].

#### Hematoxylin-eosin (HE) staining

The INIAP-IPIMAR’s routine protocol, which is based on Martoja et al. [28], was used for HE study. In summary, small tissue samples from deep layer and margins of oral arms and bell, stored in formaldehyde 4 %, were dissected and processed as following: fixation in paraffin block (Leica TP1020, Germany); formaldehyde 4 %, 1 h; ethanol 70 % 1 h; ethanol 96 % 6 h; ethanol 100 % 2 h; xylene 6 h; total 16 h. Leuckart molds were used for the inclusion of pieces in melted paraffin (58 to 60 °C). The product was cut into 5 mm-thick slices (Leica microtome SM2000 R). The application of the cuts on the slide was preceded by a water bath (20 °C) in a solution of glycerol and albumin. After applying, the blade was dried at 58 °C for at least 30 min. Staining: dewaxing, 15 min; moisturizing, 5 min; immersion solution Gill 2 hematoxylin (Sigma GHS-2, USA), 30 s; washing with water, 20 s; washing with HCl 1 % in ethanol 70 % 2 s; washing with warm water, 1 min; washing with ethanol 70 % 2 s; immersion in alcoholic solution of eosin Y withph loxine (Sigma HT110-3), 2 min; dehydration ethanol 100 %, 5 min; clarification with xylene. In HE staining, organic phosphate groups, as nucleic acids, were coloured purple; organic cationic structures, as cationic proteins of cytoplasm, were coloured red/pink.

For the other stains, samples were prepared by the smear technique. In brief, tissues from the margins of fresh oral arms were dissected to a slide and crushed with the aid of another slide. In order to benefit from the known property of adhesion to glass shown by some compounds, like plasma proteins [29], the samples were allowed to rest at at 4 °C for at least 20 min, to strengthen its grip on the slide, before the staining procedure.

#### Masson’s trichrome staining

The technique described by Kim et al. [30] was adopted with slight modifications. Weigert’s hematoxylin solution (Merck 115973, USA) – equal parts of solutions A and B – was applied over the smear for 5 min; and then gently washed with cold water (4 °C). The slide was stained with xylidine Ponceau-acid fuchsin solution – 0.1 g of xylidine Ponceau (Merck115927) and 0.05 g of acid fuchsin (Merck105231) dissolved in 100 mL of water and 0.3 mL of acid glacial acetic – for 5 min. After staining, the slide was washed with water as above and phosphomolybdic acid solution – 5 g of phosphomolybdic acid (Merck 100532) dissolved in 100 mL of water – was applied for 1 min. The slide was rewashed with water, as above, treated with the light green solution – 0.2 g of light green (Merck 115941) dissolved in 100 mL water and 0.2 mL of glacial acetic acid – for 1 min, rewashed with water and dehydrated with ethanol sequences of 70 %, 96 %, absolute and xylene. In Masson’s method, collagenous structures coloured green and organic cationic structures, as cationic proteins, coloured red.

#### Alcian blue staining

The method was adapted from Howard and Smith [31]. The solution of alcian blue (Sigma A-3157) – 0.5 g of alcian blue in water, 3 mL of glacial acetic acid and the volume was completed to 100 mL and adjusted to pH 2.5 with acetic acid (stored in the dark for no longer than 1 week) – was added over the entire sample, covering it for 1 h and then gently washed with cold water (4 °C). After that, sample was contrasted with neutral red (Sigma 861251) – 1.0 g of neutral red dissolved in 100 mL of water (stored in the dark for no longer than 1 week) – for 5 seconds and washed as above. The slide sample was then dehydrated with ethanol 96 % and ethanol 100 %, for 2 min each, and clarified with xylene for 15 min. In the technique of alcian blue, acid polysaccharides coloured blue.

#### Von Kossa staining

The method was carried out according to Howard and Smith [31] with little modifications. The smear was covered up with a solution of silver nitrate 5 % w/v and expose to intense daylight for 35 min. Then, the slide was gently washed with cold water (4 °C) and overlaid with a solution of sodium thiosulfate 5 % w/v for 5 min. The slide was washed with water as abovementioned and a solution of neutral red – 0.1 g of neutral red (Sigma 861251) and 5 g of aluminum sulfate (SigmaA7523) dissolved in 100 mL of water, stored in the dark for no longer than 1 week – was added to it for 5 s. The sample was rewashed with water and dehydrated as in alcian blue procedure. In the technique of von Kossa, inorganic phosphates form insoluble silver salts, coloured black; calcium is indirectly detected. After staining preparation, the slides were mounted with Entellan® (Merck1079610100).

#### Optical microscopy (OM)

As a first approach, the procedure followed Peach and Pitt [27]. Observations were performed in ATC2000 and DMLB Leica microscopes. ATC 2000 equipped with objective Plan Achromat 100×/1.25 (ref 13613344) and 10× wide field eyepiece (20 mm FOV) (ref 13613331). DMLB equipped with a magnification changer for DM up to 2× (ref 11505252), a 0.70× thread (ref 11541543), objective 100× (ref 11506197) and eyepiece 16x/15B (ref 10450631).

**Fig. 3:**
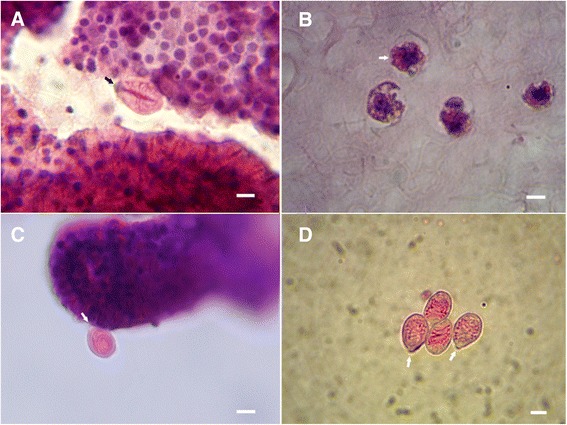
Nematocytes of *C. tagi* viewed with hematoxylin and eosin staining using optical microscopy. Purple dots indicated nucleus cells; cationic structures coloured red/pink. Arrows in (A, C, D) highlight operculum. **a** Adult eurytele surrounded by developing nematocytes. **b** Arrow points out to early stage of nematocyst morphogenesis (*orange*), developing in the gastric region. **c** Adult birhopaloid near to developing nematocytes. **d** Adult isorhizae. Bars: (**a**) 4 μm; (**b**) 3 μm; (**c** and **d**) 7 μm

**Fig. 4:**
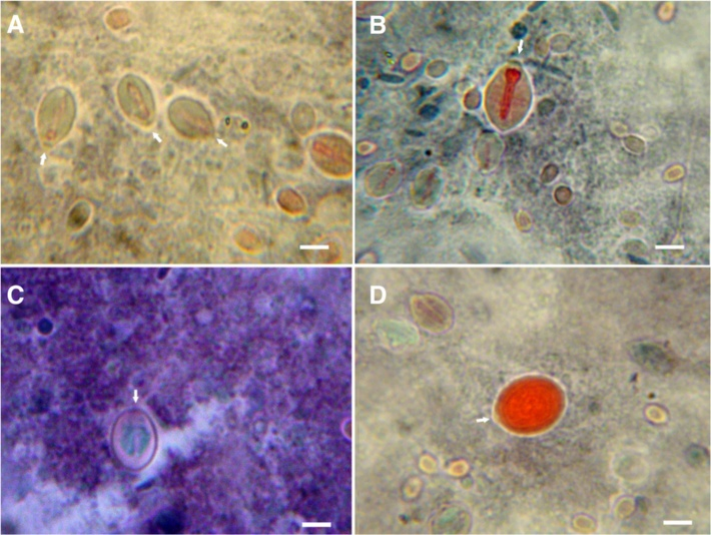
Nematocytes of *C. tagi* viewed with Masson’s trichrome staining using optical microscopy. Arrows highlight operculum. Collagenous materials in green or blue. Red colour is attributed to cationic proteins. **a** and **b** Euryteles; shaft stained in red and around it a green material filling the capsule. **c** Undefined nematocyst with a green material filling the core. **d** Birhopaloid, all stained in red due to its tubule which fulfills the inner capsule. Bars: (**a** and **b**) 5 μm; (**c** and **d**) 4 μm

**Fig. 5:**
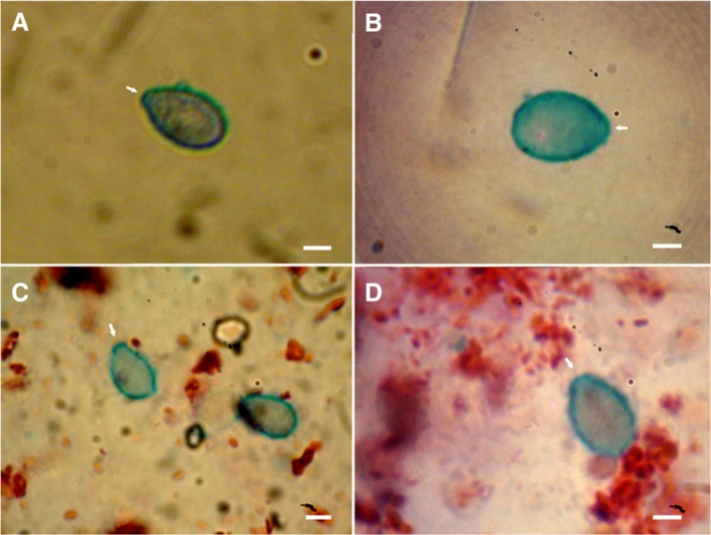
Nematocytes of *C. tagi* viewed with alcian blue staining using optical microscopy. Macromolecules with carbohydrate containing carboxyl and/or sulfate groups are stained blue. Arrows are pointing out to operculum. **a** Isorhiza type. **b** Outer wall of an undefined nematocyte. **c** Possible birhopaloids. **d** Adult eurytele. Bars: (**a** and **d**) 4 μm; (**b**) 3 μm; (**c**) 5 μm

**Fig. 6:**
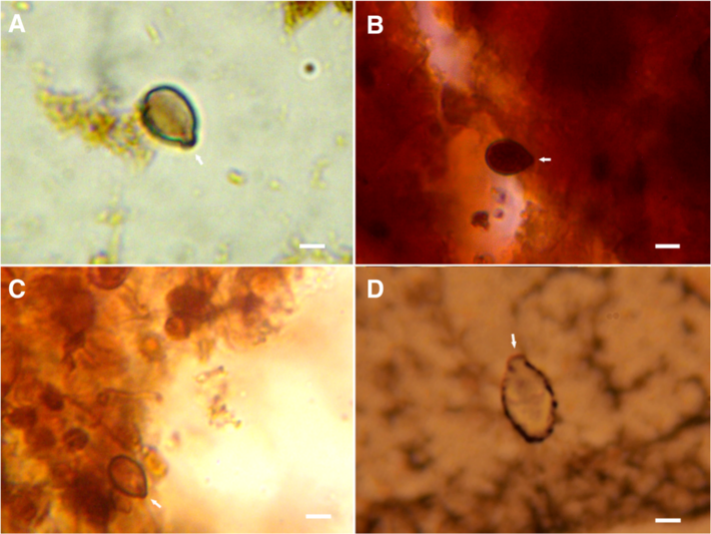
Nematocytes of * C. tagi * viewed with von Kossa staining sing optical microscopy. The black circle around or dots inside nematocytes are considered positive for calcium salts. Arrows highlight operculum. **a** and **d** Euryteles. **b** and **d** Isorhizae. Bars: (**a**) 4 μm; (**b** and **c**) 5 μm; (**d**) 3 μm

Photomicrographs were acquired both through Leica integrated system, being DMLB microscope coupled to DFC 290 HD camera and to Leica application Suite (LaS) v3.8 software, and manually, by adjusting SonyDSC-W215 camera (Japan) to the eyepiece or by adjusting a Nikon D800 camera (Japan) at the top of DMLB microscope, after removal of original Leica camera. Manual photomicrographs were edited with Microsoft Office Picture Manager v.2010 (USA) and Adobe Photoshop CS2 (USA). Figures acquisitions were as follows: Fig. 3 (a, b and d) manual, ATC 2000 and Sony DSC-W215, (C) manual DMLB and Nikon D800 (photo by Luis Quinta); Fig. 4 (a, b and d) manual, ATC 2000 and Sony DSC-W215, (C) Leica integrated system; Fig. 5 (a) manual, ATC 2000 and Sony DSC-W215, (b and d) manual, DMLB and Sony DSC-W215, (c) Leica integrated system; Fig. 6 (a and d) manual, ATC 2000 and Sony DSC-W215, (b and c) Leica integrated system.

## Results and discussion

### Relationship between *Catostylus* communities from Sado and Tagus

Macroscopic observation of the specimens, either in their natural habitats or in the laboratory, did not show relevant differences between them. In a similar way, the observations by optical microscopy indicated no significant variances in *Catostylus* samples from Tagus and Sado. Comparisons at the molecular level became possible only after the implementation of the dialysis and lyophilisation procedures which led to DNA sufficiently pure and concentrated for the next steps. The similarity of fragments from nuclear genes 18S, 28S and ITS1 rDNA for *Catostylus* samples from Sado and Tagus was respectively 97, 99 and 99 %. As a conclusion, unlike two *Cyanea* communities from the northeast Atlantic that have been recently discriminated through morphological and molecular analysis [32], it was considered confirmed that both *Catostylus* communities from Sado and Tagus do belong to the same species, named *Catostylus tagi* by Haeckel in 1869 [33]. Representative sequences of the 18S, 28S and ITS1 rDNA were deposited in GenBank with the codes KM519753, KM519754 and KM519755, respectively. The comparison of these fragments with the Australian *Catostylus, C. mosaicus*, showed identities of 97, 87 and 91 %, respectively. These differences in molecular data are in agreement with macroscopic differences like colour – *C. tagi* is cream or off-white while *C. mosaicus* is popularly known as blue blubber jellyfish – and oral arms morphology – in *C. mosaicus* the manubrium divides into four oral arms which latter bisect [34], in *C. tagi* the bisection from four to eight oral arms occurs earlier. Other comparisons will be presented later on.

### Philogenetic relationship between *Catostylus* tagi and other cnidarians according to 18S rDNA

Sequences were aligned using Clustalx v.2.0 and cladogram was automatically constructed under the maximum likelihood criterion. The cladogram presented a normal shape except that *Catostylus tagi* was found closer to *Lychnorhiza lucerna*, an Atlantic scyphozoan, than to *C. mosaicus* (Fig. 1). Results of somewhat unexpected relationship between families Catostylidae and Lychnorhizidae, among others, were already mentioned by Bayha et al. [6].

### Morphology and morphochemistry of *C. tagi* nematocytes

Size was the sole significant difference detected on the outside of the intact nematocytes by SEM method. Both the oral arms and the bell margins had nematocytes between 2.5 and 11 μm long. Figure 2a shows a representative image of the larger, reaching 10 μm in the longest dimension which contains the operculum. Examples of discharged nematocysts are shown in Fig. 2b, probably isorhiza type with atrichous tubule, being the length of tubules around 25 μm.

Considering only intact cells, three different morphological categories of nematocysts were found in specimens of *C. tagi*, euryteles, birhopaloids and isorhizae, which are common in scyphozoans as well as in hydrozoans [35], the euryteles having a broad and prominent shaft (Fig. 3a) – similar to stenoteles of *Hydra* [36], the birhopaloids presenting coiled tube around the entire nematocyte (Fig. 3c) and the isorhizae with no prominent shaft and undifferentiated tubule along the length (Fig. 3d).

In relation to the occurrence, nematocytes were more abundant in oral arms than in bell margins, although with the same relative abundance of nematocyst type. Approximate distribution was euryteles 55 %, isorhizae 30 % and birhopaloids 15 %, both in oral arms and bells. Although there were euryteles, isorhizae and birhopaloids of several sizes, the most common euryteles had an average size of about 5 μm while the isorhizae and birhopaloids were approximately 10 μm. In general, the size of a birhopaloid or an isorhiza was twice the eurytele.

Previous studies on *C. mosaicus* revealed a prevalence of isorhizae in oral arms, being usually smaller than euryteles; *Copepod nauplii*, larval form of bivalve gastropods, were the predominant prey captured by that medusa [27]. Studying the zooplankton that *C. tagi* feeds on was not planned in the present work; nevertheless, it can be mentioned that several captured specimens contained a small shrimp inside while bivalves were never found. Our observations are in agreement with Purcell [36] who reported that stenoteles are adequate nematocysts to capture crustaceans. The difference on prey selectivity between jellyfishes of the same genus may be due to the different environments they are inserted [37].

Figure 3b shows cells of *C. tagi* from deep layer of oral arms stained with HE, in which, together with the nucleus and cytoplasm, there are small circular eosinophilic structures (orange) that may be the nematocysts in early stages. This image resembles the nematocyst morphogenesis reported for *Hydra* with confocal microscopy [11]. In Figs. 3a, c, and d a neurytele, a birhopaloid and isorhizae nematocysts, respectively, are distinguishable by their interior. The intense red colour in the shaft of eurytele suggests a basic protein material. Coiled tubules of birhopaloid and isorhizae are also coloured reddish, which is consistent with the hypothesis that the tubular material is the same in the three types of nematocysts. Studies on tubules of *Hydra* have reported two basic proteins, named spinalin [10] and cnidoin [15, 17]. Since spinalin is part of the tubule spines, the more intense red colour in eurytele may indicate a double effect of spinalin and cnidoin, whereas birhpaloid and isorhizae, as they have few or no spikes, present the colour only due to cnidoin.

Figure 4 illustrates the results of Massons’s trichrome staining on the nematocytes of *C. tagi*. In order to interpret the results, it should be remembered that during the drying process different proteins will form networks with different characteristics. For example, as reported by Bancroft and Layton [38], erythrocyte protein will produce a dense network, muscle cells will form larger pores and collagen will show the least dense quite porous network. Another aspect is the general rule in trichrome staining which states that “a smaller dye molecule will penetrate and stain a tissue element but whenever a larger dye molecule can penetrate the same element, the smaller molecule will be replaced by it” [38]. In the present work, Weighert’s hematoxylin (MW ~358) was the first stain followed by xylidine Ponceau (MW ~494) with acid fuchsin (MW ~586) and the third stain was light green (MW ~793). As shown in Figs. 4a, b, d the shaft of euryteles and the tubule of birhopaloid coloured red, meaning that these structures have a cationic nature which interacted with acid fuchsin, in agreement with the previous results obtained for eosin. Figures 4a and c show green regions inside nematocytes that are attributable to collagenous material. Moreover, in Fig. 4c it is suggested that the toxin inside the nematocyst may have collagenous nature.

The images obtained for *C. tagi* with alcian blue staining showed an intense blue colour uniformly distributed around the nematocyte capsule, whether in eurytele, isorhiza or birhopaloid type (Fig. 5a, b, c, d). This result indicates the presence of a macromolecule with anionic characteristics, sulfate and/or carboxylate groups, which could be an acidic mucin (or proteoglycan), or a glycoprotein with anionic groups or a polypeptide/protein with many acidic groups (Asp or Glu). Regarding the nematocyte components already identified in other cnidarian, it seems that *C. tagi* may have a glycoprotein similar to NOWA, detected in *H. magnipapillata* [12], or to nematogalectin detected in *H. viridis, H. oligactis* and *H. vulgaris* [14] or a complex of poly-gamma-glutamate connected to chondroitin as detected in *H. vulgaris * [13]. Considering the similarity between the blue stained in *C. tagi* with that obtained by Burketová et al. [39], it is also possible that the macromolecule detected is a type of chitin, with acid character, as already mentioned in the literature of cnidarians [40] and mollusks [41]. Interestingly, Tibballs et al. [42] have postulated that this kind of macromolecule, which is a structural component of the nematocytes, “may separately trigger antigenic, allergenic or innate immune responses” in humans upon skin contact; nowadays, immunological responses to jellyfish stings are being studied, together with the typical toxinological ones.

By von Kossa staining, phosphate and/or carbonate precipitate with silver demonstrating indirectly the sites of calcium occurrence. For the nematocytes of C. tagi calcium was detected mainly associated with the outermost region, as shown in fig. 6, which could be due to the interaction of calcium with the carboxylate and/or sulfate groups of the outer membrane. Hidaka and Afuso [43] have proposed that calcium may play a role in nematocyst discharge, whether by cation exchange with a bigger divalent cation (Mg^2+^) or with two monovalent cations (K^+^ or Na^+^), to increase the internal osmotic pressure, or by “biochemical modifications of structural components such as the nematocyst stopper”. Our results seem to agree with the last hypothesis because, although there is a continuous occurrence of calcium around the whole nematocyte (Fig. 6a, b, c, d), it does not explode during discharge; on the contrary, only the operculum opens and shaft gets out without disconnecting from the whole cell which keeps its original format (Fig. 2b).

In view of the more common metals in proteins, a first attempt was carried out for the copper, through Shikata’s Orcein method [44], with inconclusive results.

## Conclusion

The optimisation of sample preparation for DNA extraction enabled further studies on jellyfish identification as, for example, other *Catostylus* including *C. cruciatus*, *C. tripterus* and * C. perezi* [33, 45], which are little known species.

The improvement of the smear procedure has simplified the use of stained reactions in gelatinous animals. Also, it was shown that good slide images can be acquired manually. Because the histochemical techniques involve affordable materials and reagents, they can be easily spread in many laboratories; however, this valuable tool requires a meticulous and delicate work, as the size of nematocyte is close to the limit of optical microscopy. The development of specific reactions, with the traditional dyes and others, can give important contributions for clarifying the nematocyte composition, together with other techniques of microscopy and proteomics. The characterisation of nematocyst toxins by staining procedures is a goal to achieve.
